# Gut Bacteria and Melamine Toxicity

**DOI:** 10.1289/ehp.121-a149

**Published:** 2013-05-01

**Authors:** Bob Weinhold

**Affiliations:** Bob Weinhold, MA, has covered environmental health issues for numerous outlets since 1996. He is a member of the Society of Environmental Journalists.

Melamine and its potential toxicity have drawn increased attention since a 2008 crisis in China in which milk, infant formula, and other foods were illegally laced with the chemical in order to increase their apparent protein content. That contamination affected an estimated 294,000 infants and children, with more than 50,000 hospitalizations and at least 6 deaths.^1^ In a new analysis of specific mechanisms possibly involved with melamine toxicity, researchers from China, the United Kingdom, and the United States have found that at least one intestinal bacterial species, *Klebsiella terrigena*, may play a role.^2^

Melamine is commonly used as an ingredient in certain laminates, coatings, plastics, resins, adhesives, dishware, kitchenware, and rubber and paper products. It also occurs as a breakdown product of the pesticide cyromazine. Melamine or substances that degrade to melamine may be illegally added to dairy products or animal feed to meet the appearance of adequate protein content. The melamine can make its way into the milk, eggs, fish, and meat of livestock given adulterated feed. It can also leach into food from adhesives used in food packaging. Occupational exposure may occur at jobsites where melamine is produced or used.^1^^,^^3^

As a member of the Proteobacteria phylum, the *Klebsiella* genus is ubiquitous in the environment and can be found in and on the intestinal tract, skin, and pharynx of humans.^4^ The limited data available for humans so far suggest its proportional occurrence in the gastrointestinal tract is on the lower end of the bacterial species that can be currently identified, but its presence is widespread and noticeable.^5^^,^^6^^,^^7^ Human microbes can vary substantially from person to person as a result of factors such as diet and geographic location.^6^

This research team found that *Klebsiella* participates in melamine metabolism and helps increase the production of cyanuric acid. This substance, in turn, is thought to contribute to the formation of stones in the kidney, ureter, and bladder—a strong suspect in the renal damage seen in melamine-exposed children.^1^ However, many of the details of the substances and mechanisms involved in this potentially harmful process remain unknown.

The team studied small numbers of Wistar rats. Different groups were fed either a high dose of melamine (600 mg/kg/day) alone for 15 days, melamine following a 4-day course of an antibiotic that killed many species of gut microbes, or antibiotic alone. The rats fed antibiotic alone or melamine and antibiotic had less, little, or no kidney damage compared with those fed just melamine, suggesting the absence or reduction of certain gut bacteria species helped reduce kidney damage. The bacterial reduction was also linked with a doubling of the urinary excretion of melamine, possibly reducing its duration in the body and potential to do harm.^2^

The team also cultured feces from young male rats and demonstrated that melamine was directly converted to cyanuric acid by fecal bacteria. They hypothesized this was a result of deamination, a process they say is “highly efficient if melamine is used as the sole nitrogen source.” But production was sharply reduced when a second nitrogen source—a soy broth—was present. This suggests that production of cyanuric acid may differ substantially among individuals (depending on variables such as diet), thus altering the relative toxicity of melamine in different people.

**Figure:**
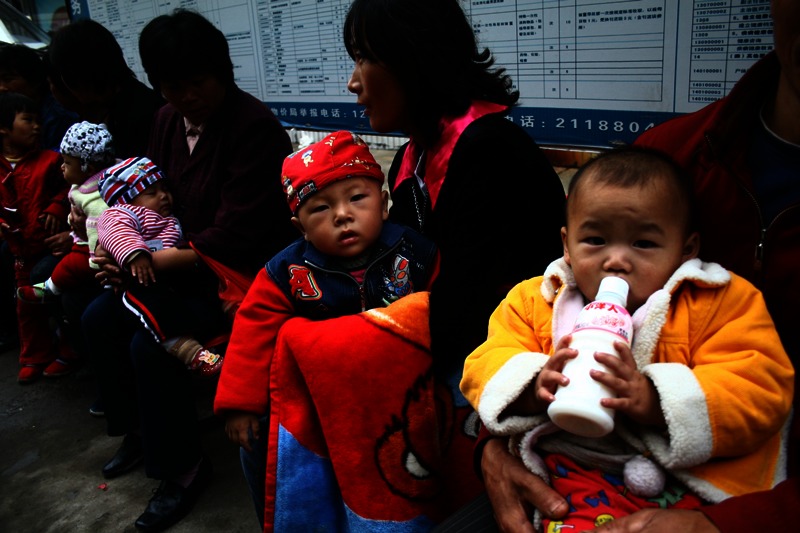
Chinese parents wait at a hospital for their children to be examined after drinking tainted milk powder, Anhui province, September 2008. Imaginechina via AP Images

Earlier research by others had found that three *Klebsiella* species, including *K. terrigena*, could play a role in cyanuric acid formation, and the team found those and four other *Klebsiella* species in the rat feces. Cultivation of *K. terrigena* in the presence of melamine showed that cyanuric acid was produced within an hour, peaked within 10 hours, and stayed steady thereafter.

In another step, the team found that orally colonizing melamine-fed rats with *K. terrigena* led to sharply and significantly increased cyanuric acid production and kidney toxicity, compared with noncolonized rats. This combined evidence led the authors to conclude that *K. terrigena* may contribute to increased melamine toxicity.

Hani El-Nezami, an associate professor of food safety and toxicology at the University of Hong Kong, raises a cautionary note about this set of studies. He says other doses of melamine, other test animals, and additional body system end points (including toxics crossing the placenta) need to be evaluated, given the variation in and hints about such factors in other recent studies.

Still, Peter Turnbaugh, a microbiologist and principal investigator at Harvard’s Faculty of Arts and Sciences Center for Systems Biology, says this particular study “provides an important proof of principle that the composition of our associated microbial communities can influence the metabolism of toxic compounds found in the diet.” He says appropriate venues for doing the necessary additional research include gnotobiotic mouse models—that is, mice tightly controlled to contain only known microbial communities—anaerobic/aerobic cell cultures, and possibly human studies.

Seppo Salminen, director of the Functional Foods Forum in the Joint Program of the Life Sciences Faculty and Medical Faculty at Finland’s University of Turku, says the methods used in this study are “appropriate and quite refined for testing this hypothesis.” He agrees that additional research is needed, in part because the mechanisms suggested in this study may well extend beyond melamine, *Klebsiella*, the intestinal tract, and kidneys. He says, “There are studies pointing out that microbial activation or inactivation of not only toxins but also of pathogens and viruses can take place on any mucosal surfaces where we always have a microbiota.”
